# Dose Adjustments of Pegcetacoplan in a Patient With Paroxysmal Nocturnal Hemoglobinuria Undergoing Surgery: A Case Report

**DOI:** 10.7759/cureus.81134

**Published:** 2025-03-25

**Authors:** Miriam Vara Pampliega, Javier Arzuaga-Mendez, Maite Moreno Gamiz

**Affiliations:** 1 Hematology and Hemotherapy, Hospital Universitario Cruces, Barakaldo, ESP

**Keywords:** complement, dose adjustment, paroxysmal nocturnal hemoglobinuria (pnh), pegcetacoplan, surgery

## Abstract

Paroxysmal nocturnal hemoglobinuria (PNH) is an acquired clonal hemolytic anemia mediated by complement that may present with thrombosis and bone marrow failure. Treatments inhibiting complement component 5, such as eculizumab, improve intravascular hemolysis and anemia and decrease the risk of thrombosis. However, anemia persists in some patients because of extravascular hemolysis. In this context, pegcetacoplan, a pegylated complement component 3 inhibitor, is a valuable treatment option for patients with PNH, as it inhibits both extravascular and intravascular hemolysis, improving anemia. Infections and surgery may trigger complement activation, and dose adjustments of treatments that inhibit complement components may be necessary in these situations to avoid episodes of breakthrough hemolysis. Here, we present a case of a 67-year-old male with PNH who, after initiating pegcetacoplan in 2022, underwent three scheduled surgeries and one emergency surgery. We report the successful use of dose adjustments and close monitoring to prevent breakthrough hemolysis during these perioperative periods.

## Introduction

Paroxysmal nocturnal hemoglobinuria (PNH) is a rare, clonal, acquired, complement-mediated hemolytic anemia that may present with bone marrow failure. PNH patients have a glycosylphosphatidylinositol (GPI) anchor protein deficiency due to somatic mutations in the phosphatidylinositol glycan class A (PIGA) gene in bone marrow stem cells. As a result, PNH patients lack two GPI-anchored proteins on the cell membrane, CD55 and CD59, whose function is complement regulation. This deficiency results in increased complement sensitivity of PNH cells, which accounts for intravascular hemolysis. The most common symptom in patients with PNH is fatigue; thrombosis and renal failure are the main causes of death [1,2]. Before the advent of anti-complement drugs, patients with PNH were treated with erythropoiesis-stimulating agents, transfusions, and immunosuppressants if PNH coexisted with bone marrow failure [3,4]. The first drugs developed specifically to treat PNH were inhibitors targeting terminal complement component 5 (C5), introduced more than 15 years ago [5]. These inhibitors improved hemolysis, anemia, and fatigue, and decreased the risk of thrombosis. However, anemia persisted in many patients due to both intravascular and, especially, extravascular complement component 3 (C3)-mediated hemolysis [6]. Inhibition of the proximal C3 component prevents both extravascular and intravascular hemolysis by blocking the complement cascade at the proximal portion of the common pathway. With this aim, the anti-C3 monoclonal antibody pegcetacoplan was developed and has demonstrated improvement in anemia in these patients [7,8]. Moreover, intercurrent infections and surgical interventions can trigger hemolytic episodes due to complement activation [9]. Dose adjustments of pegcetacoplan may be necessary to prevent hemolytic episodes during surgeries or infections; however, no established guidance or expert consensus currently exists on perioperative management in PNH patients receiving pegcetacoplan.

Here, we present a case of a patient with PNH treated with pegcetacoplan who successfully underwent major surgeries following dose adjustments and close monitoring.

This case report was previously presented as an article at the 2023 American Society of Hematology meeting, doi.org/10.1182/blood-2023-182242.

## Case presentation

**Figure 1:**
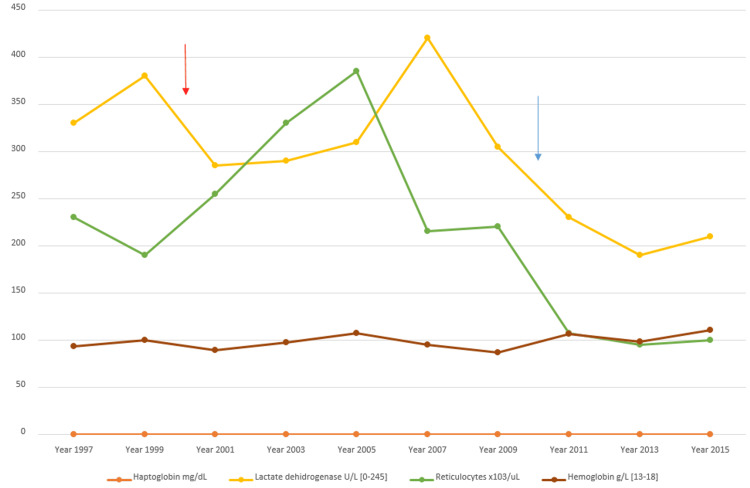
Evolution of laboratory data from 1997 to 2015 This figure illustrates the evolution of laboratory data from 1997 to 2015. The red arrow indicates the diagnosis of PNH, while the blue arrow marks the initiation of treatment with the C5 inhibitor eculizumab. After the start of eculizumab, LDH and reticulocyte levels improved; however, hemoglobin and haptoglobin levels did not show significant improvement. PNH: paroxysmal nocturnal hemoglobinuria, LDH: lactate dehydrogenase.

A 67-year-old male was diagnosed with aplastic anemia in 1997 and treated with immunosuppression. Since then, he presented with decreased haptoglobin, lactate dehydrogenase (LDH) levels above the upper limit of normal (ULN), and reticulocytosis (Figure 1). However, repeated Ham tests for PNH were negative.

In 2000, the patient suffered an intravascular hemolytic crisis for the first time, which was determined to be caused by PNH, as evidenced by a positive Ham test. Between 2000 and 2010, the patient presented with multiple hemolytic anemia crises that required red blood cell transfusions and treatment with human recombinant erythropoietin. In addition to these crises, the main symptoms experienced during those 10 years were fatigue, choluria, esophageal spasms, and erectile dysfunction. Although he had never suffered a thrombotic episode, he was treated with acenocoumarol from 2006 to 2011 as thrombosis prophylaxis [10].

In 2010, the patient initiated treatment with eculizumab due to impaired renal function following an acute hemolytic crisis. While eculizumab reduced LDH levels, anemia persisted, and the patient continued to require transfusions (Figure 1). Most of the symptoms associated with PNH improved; however, fatigue remained.

One month after starting eculizumab, the direct Coombs test (DCT) for C3d was positive. The patient remained on eculizumab, and both dose and frequency were increased in 2011, 2016, and 2017 because of pharmacokinetic breakthrough hemolysis. In 2017, the patient was receiving eculizumab at a dose of 1200 mg every 14 days; however, he still presented with anemia (hemoglobin level of 80-100 g/L) and a positive DCT for C3d.

Therefore, in December 2022, treatment with pegcetacoplan was initiated at the standard dose of 1080 mg subcutaneously (SC) twice a week. This led to an improvement in anemia (hemoglobin levels increased from 103 g/L to 130 g/L), LDH levels (from 281 U/L to 230 U/L (ULN 245 U/L)), and reticulocyte count (from 5% to 2.5%); fatigue also improved significantly.

Over the next two years, the patient underwent three scheduled major surgeries under general anesthesia. The first surgery took place in June 2023, a radical prostatectomy for symptomatic benign prostatic hypertrophy. In November 2023, he underwent the second surgery, which was a laminectomy for lumbar spinal stenosis. In January 2024, the third surgery consisted of urethrotomy and bladder neck incision to address urethral stenosis. Additionally, the patient underwent an emergency urethrotomy in April 2024 for urethral restenosis, under spinal anesthesia.

These procedures posed a significant risk of complement activation and breakthrough hemolysis, necessitating careful perioperative management. To minimize the risk of a hemolytic crisis potentially triggered by the scheduled surgeries, the dose of pegcetacoplan was adjusted in all three cases by administering 1080 mg SC every 72 hours for 10 days before and after surgery [11]. After the surgeries, LDH levels remained stable, and hemoglobin levels decreased due to intraoperative blood loss. This increased dosing of pegcetacoplan was safe and well tolerated.

**Figure 2:**
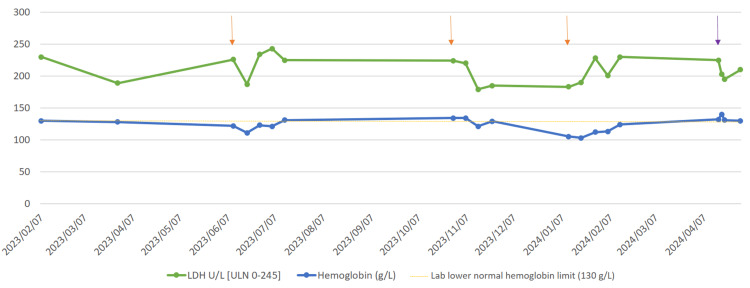
Evolution of LDH and hemoglobin values during the four surgeries performed. Evolution of LDH and hemoglobin values during the four surgeries performed. Three of them were scheduled in June 2023, November 2023, and January 2024 (marked with orange arrows). During these operations, the dose of pegcetacoplan was increased to 1080 mg every 72 hours from 10 days before to 10 days after each surgery. Urgent surgery was performed in April 2024 (marked with a purple arrow). The dose of pegcetacoplan was not increased either before or after it. In all four surgeries, LDH levels remained stable (never above ULN), and hemoglobin decreased, which was attributed to bleeding from the surgery. LDH: lactate dehidrogenase. ULN: upper limit of normal.

The emergency surgery was performed on the day the patient was scheduled to receive pegcetacoplan, so no additional doses were administered before surgery. We closely monitored hemolysis with laboratory parameters for 7 days after surgery, with no additional doses of pegcetacoplan beyond the standard regimen. Stable hemoglobin and LDH levels indicated that no further dose adjustments were required (Figure 2).

## Discussion

Historically, PNH treatment was limited to supportive care, with a median survival of 10 years [12]. The introduction of C5 inhibitors like eculizumab in 2007 marked a significant advancement, improving survival to match that of age- and sex-matched controls [13]. However, approximately 34% of patients receiving eculizumab still required blood transfusions [13].

Pegcetacoplan was the first C3-targeting therapy for PNH and was approved in 2021 by the European Medicines Agency [14]. In Spain, it is indicated for patients with PNH who remain anemic after being treated with a C5 inhibitor for at least three months. Two phase III clinical trials demonstrated the efficacy and safety of pegcetacoplan compared with either eculizumab [8] or standard of care (excluding eculizumab/ravulizumab) [15]. Despite control of both extravascular and intravascular hemolysis, patients receiving pegcetacoplan may still experience episodes of breakthrough hemolysis due to pharmacokinetic factors (incomplete complement inhibition) or complement-activating triggers, such as infections or surgeries [9,11].

Here, we present the first report of dose adjustments of pegcetacoplan in a patient with PNH who underwent three scheduled major surgeries in Spain.

To prevent a breakthrough hemolytic crisis in our patient, we considered two options: administering an extra dose of eculizumab or increasing the frequency of pegcetacoplan. In the context of scheduled surgeries, we selected the second option, taking into account the patient's previously positive experience with pegcetacoplan. This approach also avoided the need to introduce another anticomplement drug, and no published data supported the use of the first option.

The increased frequency of the perioperative dose was successful in preventing the development of a breakthrough hemolytic crisis, maintaining stable LDH levels 10 days after surgery without any complications. Additionally, no dose adjustments were made when the patient underwent emergency surgery. In that case, close postoperative monitoring allowed us to assess hemolysis and conclude that no treatment changes were required.

Our findings highlight the need for perioperative dose adjustments to prevent breakthrough hemolysis, particularly in high-risk surgical settings.

## Conclusions

To our knowledge, this is the first report of pegcetacoplan dose adjustments in a PNH patient undergoing multiple surgeries. Our case successfully demonstrates that increasing the dose to 1080 mg every 72 hours for 10 days before and after surgery effectively prevents breakthrough hemolysis, as evidenced by stable LDH levels. However, we believe that after surgery, strict monitoring of LDH and hemoglobin should be performed, and extra doses of pegcetacoplan should be considered only in cases of active hemolysis. Further studies and case reports are needed to establish standardized perioperative protocols for PNH patients receiving pegcetacoplan. These findings should inform expert consensus statements and clinical guidelines to optimize surgical outcomes in this population.
